# Prehabilitation in Patients before Major Surgery: A Review Article

**DOI:** 10.31729/jnma.7545

**Published:** 2022-10-31

**Authors:** Pawan Shakya, Sagar Poudel

**Affiliations:** 1 Department of Surgery, Ramechhap District Hospital, Ramechhap Bazaar, Ramechhap, Nepal

**Keywords:** *length of stay*, *mental health*, *nutrients*, *preoperative exercise*, *smoking cessation*

## Abstract

The overall outcome of the patient after any surgery is determined not only by the fineness of the surgical procedure but also by preoperative conditioning and postoperative care. Prehabilitation decreases the surgical stress response and increases the preparedness of the patient to undergo planned surgical insult. Preoperatively structured inspiratory muscle exercises, cardiopulmonary fitness program, and planned exercise program for muscles of limbs, back, abdomen, head, and neck allow an overall upliftment of the physiological capacity of the patient to better cope with the surgical stress. Optimization of dietary status by macronutrients, micronutrients, and the nutrients has an impact on augmenting postoperative recovery and shortening the overall hospital stay. Preparing patients for the scheduled surgery and initiating alcohol and smoking cessation programs overhaul the patient's mental health and boost the healing process. This concept of prehabilitation a few weeks before surgery is equally beneficial compared to enhancing operative procedures and postsurgical care.

## INTRODUCTION

Prehabilitation is a tactic of enhancing the general health and wellbeing of the patient preoperatively to modify the probable risk factors and thus uplifting the physiological reserve and decreasing the adverse stress response.^1^ Though surgical interventions are indicated for the cure or palliation of various diseases, the surgery itself acts as a stressful event and thus has a deep impact on performance and quality of life postoperatively.^2^ In spite of advancements in anaesthesia, surgical procedures and perioperative care, most patients don't gain rapid functional and physiological recoveries. Contrary, the rise in life expectancy of most elderly patients has led to an increase in surgical treatments. Studies on adjustable risk factors have identified some protective elements for surgical complications and postoperative recovery, which include good physical fitness, functional reserve, and nutrition.^34^ These preoperative factors are vital to counter the expected stress linked with surgery.

## ENHANCED RECOVERY AFTER SURGERY AND PREHABILITATION

While earlier prehabilitation intercessions engrossed only in uplifting the physical function of patients, it now incorporates the psychological ability of the patient to downgrade the potentially detrimental effects of a significant surgical stressor as well.^34^ The addition of this program with enhanced recovery after surgery (ERAS) programs allow patients for enhancing postoperative surgical outcome.^5^ With the effect on aerobic capacity and physical fitness, it has a positive interference on 30-day hospital readmissions.^6^'^7^ Prehabilitation is complementary to ERAS as it adjusts postsurgical outcomes by pursuing a preoperative physiological reserve facility, which in sequence impacts the stress response and the end consequences.^8,9^ An exaggerated systemic inflammatory response during any major surgery endorses lean muscle loss, imbalanced haemostasis, and decreased aerobic capacity.^10^

## PREOPERATIVE ASSESSMENT

Preoperative cardiorespiratory fitness reckons a person's ability to provide and use oxygen to do work, which results from a mixture of training status and genetic disposition.^11^ Numerous variables such as peak oxygen consumption, ventilatory equivalents for carbon dioxide clearance, and anaerobic threshold are associated with postoperative outcomes. General physical constraint due to inactivity and/or intolerance to exercise from certain pathology may be accountable for this.^10^

Frailty assessment measures an old patient's strength, energy, cognition, health status, functional waning, and its impact on daily activities. The Canadian study of health and ageing frailty index (CSHA-FI) has been endorsed in the geriatric population to predict the risk of death and prolonged admission. It is a judgement-based 9-point scale tool to monitor frailty and stratify degrees of fitness and frailty. It condenses information from a clinical meeting with an elder patient and roughly quantifies the patient's overall health status.^12^ There is no gold standard assessment for frailty, particularly among older individuals undergoing surgery. These measuring tools differ in the areas assessed (i.e., reasoning, comorbidities, and physical function), source of information (i.e., direct evaluation, self-report, and electronic health data), the time involved, and place of evaluation (i.e., outpatient, inpatient, and by phone. The two physical measurements in the Physical frailty Phenotype (PFP), gait speed and handgrip strength require training to standardize measurements. The second context for defining frailty is contemplating frailty as a build-up of deficits through functional, physical, reasoning, and social measures. Various tools are developed to derive a cumulative score from factors such as nutrition, comorbidities, functional status, disability, and mental health.^13^

Smoking is a recognized perioperative risk factor that affects up to one-quarter of surgical patients. Its detrimental effects on cardiac, respiratory, and immune function contribute to the patient's meagre postoperative outcome.^14^ Most of the people planning for surgery who have a presurgical history of smoking, asthma, chronic obstructive pulmonary disease, or other respiratory ailments, have all been related to a postoperative respiratory complication. Preoperative abstinence of 4-6 weeks results in a significant decline in postoperative complications.^15^ Above two units daily, alcohol appears to aggravate the neuroendocrine response to surgery leading to dose-related perioperative complications. Perilous alcohol consumption affects around one-quarter of patients undergoing surgery.^16^ Thus, curtailing consumption to within recommended limits lessens the occurrence of those complications.^17^ In one of the recent studies, 'risky drinking' is defined as any alcohol ingestion corresponding to more than 3 alcohol units (AU)/day or 21 AU/week (with 1 AU equating to 12 grams of ethanol) with or without symptoms of alcohol abuse or dependency.^18^ For an intake of more than 2 to 3 AU/d (28 grams/unit per day), the postoperative complication rate is augmented by about one-half. The complication rate for patients drinking more than 5 AU/d is increased thrice.^19^

An anthropometric assessment including weight, height, and waist circumference, body mass index, and body composition assessment are vital elements in the appraisal of patients' nutritional status.^20^ Various studies have demarcated severe nutritional deficiency as per the European society for clinical nutrition and metabolism (ESPEN) guidelines: weight loss >10-15% in past 6 months, BMI <18.5 kg/m^2^, subjective global Assessment (SGA)= c, nutrition risk screening (NRS-2002) >5, or albumin <30 g/l.^21^ Nutrition that is anticipated to be less than one half of patient's daily dietary total must be augmented pre-operatively and continue post-operatively. If enteral means do not meet energy requirements, then parenteral nutrition can be applied. As such preoperative supplementation of 10 days has been shown to decrease complications.^22^ Other secondary methods of nutrition status involve the evaluation of strength and function.

Malnutrition provokes defensive mechanisms that lessen basal metabolic rate and weakens physical performance to preserve nutrient reserves. Using a standard etiquette to ascertain handgrip, gait speed, 6-min walk test, timed up and go, short physical performance battery, and 30-s sit-to-stand are other measures for assessing the physical fitness status of the patient.^23^ Other preoperative nutritional status assessment comprises poor oral intake, insufficient protein energy ingestion, compromised nutrient consumption, altered gut function, inadvertent loss of weight, and underweight. Various additional tools have been authorized for use on admitted patients, such as the malnutrition universal screening tool (MUST), short nutrition assessment questionnaire (SNAQ), and the malnutrition screening tool (MST) but there is no undisputed screening tool for use in the preoperative "at risk" surgical patients.

Overall stressors which encompass diagnosis, surgical procedure, anaesthesia, pain, survival, and recovery, are all causes of worry and anxiety for the patient which ultimately affects postsurgical recovery by varied methods. Eventually, these preoperative stressors slower healing via innumerable psychological and immunological processes.^24,25^ Psychological factors like anxiety, depression, and poor self-esteem continually worsen physiological haemostasis and quality of life postoperatively.^26,27^ hospital anxiety and depression scale (HADS) covers two subscales i.e., anxiety and depression, each having seven items and scored from 0 to 3. A score greater than 8 on each of these subscales indicates mood disorders.^28^ Comprehensive preoperative clinical review should also be emphasized when risk factors, such as smoking, diabetes mellitus, anaemia, and other comorbidities, can be identified and controlled.

## PREOPERATIVE INTERVENTION

A combined aerobic and resistance exercise program should state the rate, strength, time, and type of activity to be performed. These exercises include walking, cycling, and swimming, which are dependent upon the patient's convenience, capacity, and preference. Other muscle-strengthening exercises should be followed using suitable equipment and under the guidance of a physiotherapist or a physical educator, a minimum of two days a week. It should aim to the reinforcement of all muscle groups aiding daily life activities which include muscles of the chest, abdomen, back, upper, and lower limbs.^29^

Preoperative inspiratory muscle training (IMT) is a form of resistance training for the respiratory muscles using a handheld device. It involves five to seven controlled sessions each week, which lasts 15-30 min, for 2 weeks before surgery. In each setting, patients breathe in a pre-defined percentage of their maximal inspiratory strength (Pimax) via an inspiratory threshold-loading device. These sessions are discontinued postoperatively as pain and other effects of surgery make this manoeuvre virtually unbearable.

Nicotine-dependent patients who generally smoke within 30 min of walking in the morning must be considered nicotine replacement therapy. This therapy should ideally be stopped a day before surgery, particularly for microvascular reconstructive procedures. Lastly, pharmacological support with varenicline or bupropion should be considered.^30^ Five sessions or more of respiratory rehabilitation with 30 min of physical activity per week are essential, with approx. 50% of these were under the guidance of a healthcare worker and continued for a minimum of 6 weeks. Alcohol cessation intervention programs vary in intensity, duration, and frequency. These incorporate reassurance, communication and suggestions, treatment of alcohol withdrawal, relapse prophylaxis endorsed by medicinal approaches, and follow up.^19^ These sessions take 4 to 8 weeks and intend to achieve complete alcohol cessation before surgery.

## NUTRITIONAL OPTIMIZATION IN PREHABILITATION

Successful nutritional intervention is carried out in a timeline beginning before surgery and further extending perioperative and postoperative periods.^31,32^ The recent guidelines by the European society for clinical nutrition and metabolism (ESPEN) fittingly show the prognostic guidance of nutritional status on postsurgical complications and mortality.^33^ Postoperative patients with malnutrition have significantly greater postoperative morbidity, deaths, duration of hospital stay, rate of readmission, and increased hospital costs. A higher body mass index (BMI) is apparently linked with the possibility of micronutrient deficiencies. While younger patients are more prone to develop nutritional deficiencies, the female gender is substantially related to iron deficiency, but numerous gender variations were observed depending on the type of nutrient deficiency.^34^

Nutritional conditioning should usually be initiated at least 4 weeks before surgery. Though carbohydrate loading one day before surgery was also found to be unproductive with regard to the duration of hospital stay and postsurgical lethargy, it does halve insulin resistance in the postoperative phase.^35^ The exercise and nutrition programs commenced between 2 and 12 weeks preoperatively have shown to increase the overall benefits. In the case of high-risk individuals with severe malnutrition, enteral nutrition should be exclusively favoured if more than 60% of the energy demand can be fulfilled. Otherwise, partial, or total parenteral nutrition may be included both preoperatively and postoperatively.^36^

Appropriate nutrition entails enough protein for anabolism which can sustain body weight in circumstances of significant surgical stress. In healthy adults, the suggested protein intake is 0.8 g/kg/day, but that may increase up to 1.2 gm to 1.5 g/kg/day for surgery patients. They are advised for daily consumption of two protein portions, ranging from 20 to 40 gm.^21,33^ Various studies recommend patients consume protein or supplements during the anabolic window i.e., up to one hour after commencement of physical exercise during which muscle protein synthesis is at its peak.^37^ Similarly adding carbohydrates a few hours before physical activity raises muscle and liver glycogen, enabling physical exercises planned per prehabilitation. It is recommended that at least 140 gm of carbohydrate three hours before working out ease physical performance, and 10 gm of protein after exercise enhance active muscle strength by 25%. Lastly, non-protein components of the diet, which include fat, carbohydrates, fibres, and micronutrients, must also be suitably assimilated.^38,39^

Insufficiency of micronutrients such as zinc, selenium, vitamin B6 and vitamin D adversely impacts the immune response. Beneficial antioxidants such as vitamins, minerals, and nutrients such as carotenoids, vitamins A and C, selenium, bioflavonoids, and glutathione can neutralize unstable free radicals stemming from trauma or surgery, thus avoiding further harm.^34^ Some authors back immunonutritients that act by modifying the inflammatory response and thus counter postsurgical immune impairment. These immunonutritients include glutamine, arginine, omega-3 polyunsaturated fatty acids, and nucleotides.^40^ Arginine, glutamine, nucleotides, and omega-3 fatty acids are the most explored immunonutritients. Arginine is a non-essential amino acid and has a role in the production of nucleotides, proline, nitric oxide, and polyamines. It also promotes lymphocyte function and enhances wound healing. Another amino acid glutamine is fuel for swiftly dividing cells in the small and large intestines. Enteral feeds with omega-3 fatty acids not only reduce proinflammatory mediators but also reduce infections.^40^

## COMORBIDITY AND RISK FACTOR OPTIMIZATION

Preoperative anaemia is related to the surge of postsurgical morbidity and is associated with an increased rate of blood transfusion in surgeries with moderate or severe blood loss.^41^ Blood transfusion is shown to have immunosuppressive effects and, even a transfusion of a single unit of packed red blood cells has been linked with increased postsurgical morbidity and mortality.^41,42^

The goal of preoperative anaemia should be to accomplish haemoglobin levels above 13 g/dl. It should be managed with oral or intravenous iron. Oral iron should be administered 40 to 80 mg daily, but intravenous iron should be started if there is intolerance to oral administration or in conditions where surgery is planned for less than 6 weeks after a diagnosis of iron deficiency.^43^

Preoperative glycated haemoglobin has been suggested as a biological prognostic marker in operating patients, and an HbA^1^c between 6% and 7% is associated with higher risks of anastomotic leaks, wound infections, major complications, and overall postoperative complications.^44^

## INTERVENTIONS FOR PSYCHOLOGICAL WELLBEING

The intervention for psychological well-being is directed to attenuate pre-operative stress and anxiety via training in various stress management methods.4 Psychoeducation targets providing patient information regarding the surgical technique, management, expected untoward effects, recovery course, functional adjustments, etc. by written resources, videos, or with the help of medical supervision. It helps to allay the anxiety levels in the patients who are apprehensive about the planned medical procedures.^45^ Preoperative cognitive intercessions emphasize the prompt documentation and handling of disorderly presurgical views and anticipations to test them. Meditation and hypnosis help mitigate stress and ameliorate the coping capacity of the patients. Several studies have found that using hypnosis before surgery lessens:

1. Use of perioperative anaesthetic agents and postoperative pain medications.
2. Postsurgical side effects include pain, nausea, vomiting, and fatigue.
3. Mood disorder, anxiety, depression, and emotional lability after hospital discharge; and
4. Dose of medication.

Integrated therapeutic modalities include a combination of at least two of the above-mentioned groups of interferences. In various studies when such integrated approaches were employed before surgery, they found:

1. Decreased levels of psychological stress.
2. Improved mood states preoperatively and throughout the following year post-surgery.
3. Promptness to use mental health services if needed.^46^

## THE IDEAL DURATION OF SURGICAL PREHABILITATION

Any duration of less than 2 to 4 weeks seems to be ineffective, similarly, more than 3 months has the drawback of poor patient adherence. Thus, the ideal time frame for a prehabilitation program should be estimated by the best correlation between program adherence and efficacy. Hence, the optimal range of prehabilitation should be from 4 to 8 weeks if the underlying disease permits.^27^ A study concluded that the effect of 12 weeks of combined upper and lower body high-intensity interval training has little but clinically relevant beneficial effects on muscular and cardiorespiratory fitness in older patients.^47^

## RECENT STUDIES ON THE BENEFITS OF PREHABILITATION

Many recent systematic reviews have studied isolated prehabilitation programs which solely used physical activity interventions, nutrition optimization, micronutrition or psychological optimization. Extents of functional capacity and cardiopulmonary testing are also vital to ascertain improvement in physical fitness and functional capacity after prehabilitation programs. Psychological and quality-of-life re-evaluations via the use of authenticated questionnaires can help identify patients and programs with the greatest benefit.^48,49^

Immunonutrition reduced wound infection by 29% (RR 0.71, 95% CI 0.51 to 0.99, I2 =6%). Smoking cessation reduced wound infection by 72% (RR 0.28, 95% CI 0.12 to 0.64, I2 =12%).^5^ According to a recent systematic review, that included seven articles and a total of 1377 patients in the quality analysis, a trend toward fewer complications and a shorter hospital stay was seen in the prehabilitation group.^50^ In a recent meta-analysis, a significant reduction in overall (OR 0.63 95% CI 0.46-0.87 I2 34%, p= 0.005) and pulmonary morbidity (OR 0.4 95% CI 0.23-0.68, I2= 0%, p= 0.0007) was observed in the prehabilitation group.^51^

According to a recent study, reducing alcohol use from four to two standard drinks per day decreases the incidence of perioperative complications rate by up to 50%.^17^ Similarly, protracted bleeding time and surgical stress responses may be overturned by 4 weeks of abstinence.^1,17^ In a study done in Montreal, 80% of patients who had received multimodal prehabilitation prior to colorectal cancer resection surgery have recovered their baseline functional capacity by 8 weeks post-surgery, compared to only a 40% recovery rate in a historical control that had received only postoperative rehabilitation.^52^ The success of surgery does not depend merely on the procedure itself, but on how quickly the patient is able to return to his or her physical and psychological state of health.^51^

## WAY FORWARD

The prehabilitation programs should be patient-centred and should emphasize factors like the type of surgery, the current state of health, and disease. The evolving model of surgical prehabilitation encompasses a multifactorial and interdisciplinary methodology. All the patients should be tactically primed for major surgeries as the preoperative period is the vital link to ultimate treatment methods and avoidance of adjustable risk factors. For this purpose, creating reproducible approaches and describing homogenous outcome breakdown tools strengthen the base for a patient-centred prehabilitation program.
